# Women’s knowledge, attitude, and practice of breast self- examination in sub-Saharan Africa: a scoping review

**DOI:** 10.1186/s13690-020-00452-9

**Published:** 2020-09-22

**Authors:** Roseline H. Udoh, Mohammed Tahiru, Monica Ansu-Mensah, Vitalis Bawontuo, Frederick Inkum Danquah, Desmond Kuupiel

**Affiliations:** 1 Faculty of Health & Allied Sciences, Catholic University College of Ghana–Fiapre, Sunyani, Ghana; 2 Research for Sustainable Development Consult, Sunyani, Ghana; 3 Department of Public Health Medicine, School of Nursing and Public Health, University of KwaZulu-Natal, Durban, South Africa

**Keywords:** Breast cancer, Breast self-examination, Self-breast examination, Knowledge, Practice, Attitude, Women, Sub-Saharan Africa

## Abstract

**Background:**

Breast cancer (BC) is a non-communicable disease with increased morbidity and mortality. Early detection of BC contributes to prompt linkage to care and reduction of complications associated with BC. Breast self-examination (BSE) is useful for detecting breast abnormalities particularly in settings with poor access to healthcare for clinical breast examination and mammography. Therefore, we mapped evidence on women’s knowledge, attitude, and practice of BSE in sub-Sahara Africa (SSA).

**Methods:**

We conducted a systematic scoping review using Arskey and O’Malleys’ framework as a guide. We searched PubMed, Google Scholar, CINAHL, and Science Direct databases for relevant studies on women’s knowledge, attitude and practice on BSE. Studies included in the review were from SSA countries as defined by the World Health Organization published from 2008 to May 2019. Two reviewers independently screened the articles at the abstract and full-text screening guided by inclusion and exclusion criteria. All relevant data were extracted, and a thematic analysis conducted. The themes were collated, and a narrative summary of the findings reported.

**Results:**

Of the 264 potentially eligible articles identified from 595,144, only 21 met the inclusion criteria and were included for data extraction. These included studies were conducted in 7 countries of which 11 were conducted in Nigeria; two each in Ethiopia, Ghana, Cameroon, and Uganda; and one each in Kenya and Sudan. Of the 21 included studies, 18 studies reported evidence on BSE knowledge and practice; two on only knowledge; one on only practice only; and six presented evidence on women’s attitude towards BSE. The study findings suggest varying knowledge levels on BSE among women in SSA countries. The study findings also suggest that BSE practice is still a challenge in SSA.

**Conclusion:**

There is a paucity of published literature on women’s knowledge, practice, and attitude of BSE in SSA. Hence, this study recommends further studies on knowledge, practice, and attitude of BSE, to identify contextual challenges and provide evidence-based solutions to improve women’s knowledge, practice, and attitude of BSE in SSA.

## Background

Breast cancer (BC) has been described as the most commonly diagnosed cancer in women and the leading cause of cancer death globally [1]. In 2018, of the 8.6 million new cases of cancer globally, BC accounted for 24.2% of which 8.1% occurred in SSA. BC also accounted for nearly 15% of the 4.2million mortality due to cancer worldwide with SSA accounting for 11.8% [1]. It is estimated that 1 in 8 women will develop BC over a lifetime and in the next decade 19.7 million new cases are expected globally by 2020, and 10.6 million will occur in low-and-middle-income countries (LMICs) [2, 3]. Similarly, it is projected that 43.1% of women will die due to BC worldwide and 36.8% will occur in LMICs by the end of 2020 [2].

These regional projections of BC incidence and mortality are worrying. Hence, demand immediate action to prevent and detect BC early through the different screening methods, as a mandate to help achieve the agenda for sustainable development goal (SDG) 3.4 by 2030 [4]. To facilitate early detection of BC, knowledge, attitude, and practice on the screening methods are essential. Although clinical breast examination and mammography are ideal for BC diagnosis, access to healthcare in most SSA countries may be a major challenge. Economic constraints in most SSA countries may impede the availability of mammography in the majority of the health facilities [5–7]. In addition, both clinical breast examination and mammography require expertise, specialized equipment, and a visit to the health facility [7]. However, breast self-examination (BSE) is a non-invasive procedure performed by the individual monthly to determine a normal breast and recognize any change on the breast for early medical care to be sought [8, 9]. Evidence shows that nine out of the ten breast lumps are detected by the women themselves [9].

To this end, knowledge, attitude, and practice of BSE among women are essential. Knowledge of BSE involves having information on signs of BC, BSE procedures and how to perform BSE [10–13]. Evidence shows that having knowledge of BSE has a positive impact on early detection of BC [14]. Knowledge of BSE may also influence the attitude and practice of BSE [13, 15]. Attitude is a settled way of thinking about BSE which includes acceptance that BSE is necessary, all women should perform it, ready to encourage other people to get information and to practice it and seeking early medical care with any abnormalities [10, 13, 16]. The practice of BSE involves the act of palpating one’s breast monthly, just after menstruation, and the ability to detect abnormalities [10, 13, 17]. The practice of BSE makes the individual becomes familiar with the structure of her breast and be responsible for her health since the detection of any abnormality will necessitate seeking early medical care [5, 18]. Despite this, to date, no study has methodically explored and described literature and identified research gaps on knowledge, attitude, and practice of BSE for future studies in SSA to the best of our knowledge. This current study, therefore, aimed to systematically map literature and describe the evidence on women’s knowledge, attitude, and practice of BSE in SSA.

## Methods

We employed Arksey and O’Malley’s and Levac et al. recommendations [19, 20] to conduct a systematic scoping review focused on women’s knowledge, attitude, and practice of BSE in SSA. The Arksey and O’Malley framework included in this study are as follows: identifying the research question, retrieving relevant studies, selection of studies, charting data, and collating, summarizing, reporting evidence. A detailed description of this study’s methodology has been previously reported in the published protocol [21]. The preferred reporting items for systematic reviews and meta-Analyses extension for scoping reviews (PRISMA-ScR) checklist was followed to report this study (Supplementary file 1).

### Identifying the research question

The main review question was: What evidence exists on women’s knowledge, attitude, and practice of BSE in SSA?

The Sub review questions were as follows:

1. What is the evidence on the knowledge of BSE among women in SSA?
2. What is the evidence on the attitude toward BSE among women in SSA?
3. What is the evidence on BSE practice among women in SSA?

### Literature search

An exhaustive search for potentially eligible articles was conducted in the following databases: PubMed, CINAHL, Google Scholar, and Science Direct to obtain relevant articles. The database search occurred in May 2019 using the following keywords: “women”, “female” “self-breast examination”, “breast self-examination”, “knowledge”, “attitude”, “practice”, “breast”, “cancer”, “breast cancer”, “Africa”, “sub sahara africa”, south of the sahara”, and “SSA”(Supplementary file 2). Boolean terms (AND/ OR) were used to separate the keywords. We also included Medical Subject Heading (MeSH) terms during the keywords search in the databases. To widen the scope of the search and capture the full range of literature on KAP of BSE, language and study design restrictions were removed during the databases search but the search date was limited from 2008 to the search date in 2019. We further search the reference list of all the included studies for eligible articles.

### Study selection

Guided by the eligibility criteria, RHU conducted database search and title screening. RHU and MT independently screened the abstracts and full articles in parallel. The discrepancies in the investigator’s responses at the abstract screening stage were discussed by the review team until a consensus was reached. However, DK resolved the discrepancies between RHU and MT at the full-text screening stage. Then, Cohen’s kappa coefficient, κ statistic between the reviewers was calculated after the full-text screening. This study included primary studies: conducted in SSA, published between 2008 to May 2019, reporting evidence among women 18 years and above, presenting evidence of women’s knowledge, attitude, and practice of breast self-examination, and published in English. However, studies conducted in other countries outside SSA, articles reporting evidence among men, studies focused on cost-effective of BSE, articles presenting evidence on CBE as well as articles presenting evidence on a mammogram, and other review articles were excluded.

### Charting the data

A thorough reading of the included studies for data extraction of bibliographic details, study title, aim/objectives, study design, target population, study setting, significant findings of interest were extracted. Other information like geographical location (urban or rural), country of study, sample size, and conclusion were also extracted to answer this review question. To ensure consistency and reliability of this study findings, RHU and DK independently extracted all relevant data from the included studies using a piloted form designed in Microsoft word.

### Collating, summarizing, and results

All relevant data extracted were analysed thematically. The themes were collated, and a summary report of the finding presented narratively with a focus on this study outcome of interest (knowledge, attitude, and practice).

## Results

**Fig. 1:**
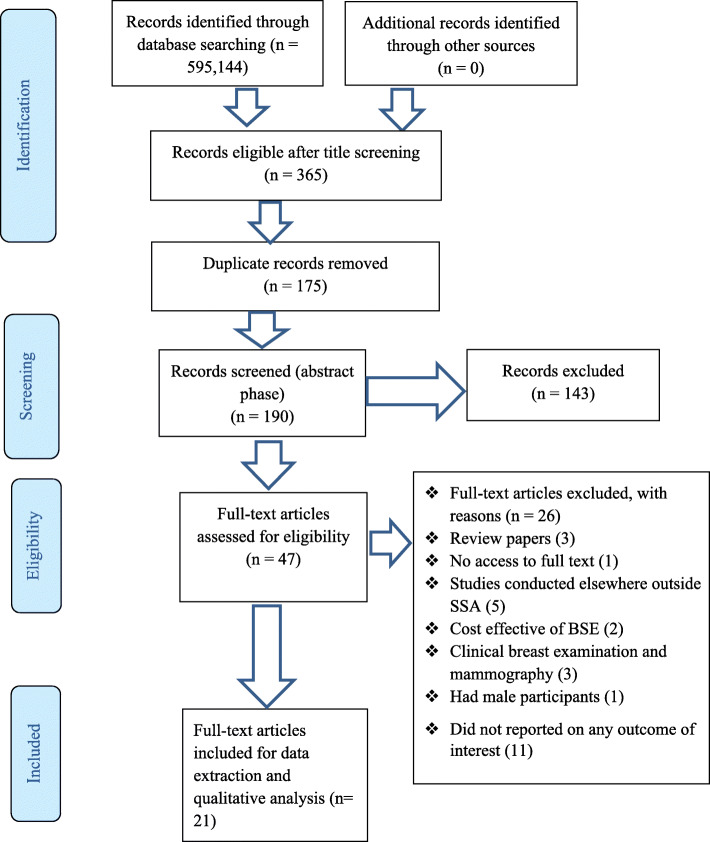
PRISMA 2009 Flow Diagram

In all, 365 articles met the eligibility criteria out of 595,144 at the title screening stage. Out of the 365 articles, 175 were duplicates hence, they were deleted prior to abstract screening. Subsequently, 143 and 26 articles were also excluded following abstract and full-text screening respectively (Fig. 1). There was a significant level of agreement between the investigators” responses at full article screening stage (Kappa statistic = 0.80, *p* < 0.01). Of the 26 full-text articles excluded, eleven did not report on any of this study outcome of interest [22–32]; five studies were conducted outside this study setting [8, 33–36]; three reported on CBE and mammography [37–39]; three were review articles [40–42]; two studies reported on cost-effective of BSE [5, 7]; one study reported evidence on male [43] and one full text could be accessed [44] despite several emails to the authors requesting for it. At the end of the study section process, 21 articles met the inclusion articles were included for data extraction.

### Characteristics of included studies

Of the 21 included studies, one was a pre-experimental study [45], two were mixed methods studies [46, 47], and eighteen were descriptive cross-sectional surveys [48–65]. The majority (11/21) of the included studies were conducted in Nigeria [46, 49–54, 57, 60, 62, 63] and the rest in Sudan [65], Kenya [64], Uganda [47, 59], Cameroon [55, 61], Ethiopia [45, 56], and Ghana [48, 58]. A total of 18 studies were conducted in urban settings [45–48, 50, 51, 53–62, 64, 65] while three were in a rural setting [49, 52, 63]. Table 1 presents a summary of the characteristics and findings of the included studies.

**Table 1:** Characteristics and study findings of included studies

Author and date	Country	Geographical area	Study setting	Study design	Study population	Significant study findings
Abera et al, 2017 [45]	Ethiopia	Urban	University campus	pre-experimental study	1^st^ year midwifery students	Before intervention 0% never detect lump after intervention 77% detected lump.
Agbonifoh, 2016 [54]	Nigeria	Urban	University campus	A descriptive survey	Female students in the tertiary institution	The course of study and knowledge of BSE significantly influenced the practice of BSE, but parental and family history of BC did not.
Casmir et al, 2015 [62]	Nigeria	Urban	University campus	A descriptive cross-sectional survey	Female undergraduate students	A statistically significant relationship between knowledge of risk factors for BC, source of information on BSE, the age of the respondents and practice of BSE.
Faronbi & Abolade, 2012 [63]	Nigeria	Rural	Senior High School	Descriptive cross-sectional survey	Female secondary school teachers	22% understood BSE helped in early detection of BC.
Fondjo et al, 2018 [48]	Ghana	Urban	Senior High School/ University campus	A cross-sectional study	Female students	More tertiary students perform BSE than SHS students. 76.3% perform BSE because of the benefits.
Godfery et al, 2016 [59]	Uganda	Urban	University campus	Cross-sectional study	Female students	38% of those not practicing planned to practice.
Gwarzo et al, 2009 [46]	Nigeria	Urban	University campus	Mix method [quantitative/qualitative]	Female students	Practice of BSE higher among those with a family history of BC. Two students had detected a lump in their breast.
Idris et al, 2013 [65]	Sudan	Urban	University campus	A descriptive cross-sectional study	Final year female medical students	46.5% performed BSE correctly and 44% found lump by performing BSE.
Isara & Ojedokun, 2011 [60]	Nigeria	Urban	SHS campus	A descriptive cross-sectional study	Female students	31.4% who had good knowledge of BSE had practiced BSE.
Kimani & Muthumbi, 2008 [64]	Kenya	Urban	University campus	A cross-sectional descriptive study	Female students	No significant difference between the preclinical and clinical in the practice of BSE.
Makanjuola et al, 2013 [52]	Nigeria	Rural	Ala community	A descriptive cross-sectional study	Women living in Ala community	34% recognize BSE as BC preventive measure.
Nde et al, 2015 [61]	Cameroon	Urban	University campus	Cross-sectional descriptive study	Female undergraduate students	Significant association between knowledge, attitude and the tendency to practice BSE. Among those who performed regularly 22.2% detected abnormal pains, abnormal lump 11.1%, discharge of pus from nipple 11.1%, and abnormal size 11.1%, 44.4% other abnormalities.
Obaikol et al, 2010 [47]	Uganda	Urban	University campus	Mix method	Female students	4.8% found to have breast lumps, 43.3% knew BSE as a screening method and 42.1% for diagnosis purposes.
Obaji et al, 2013 [51]	Nigeria	Urban	Market	A cross-sectional descriptive study	Women	38.9% of the women admit BSE as a means of early detection of BC, awareness of BSE is associated with the level of education.
Oladimeji et al, 2015 [53]	Nigeria	Urban	Markets	A descriptive cross-sectional study	Market women	Knowledge of the performance of BSE increased with age and marital status.
Olowokere et al, 2012 [49]	Nigeria	Rural	Health facility	A descriptive cross-sectional study	Rural women	Majority of the women [61.1%; n= 110] who were not practicing BSE likely to start practicing it.
Onwere et al, 2009 [57]	Nigeria	Urban	Antenatal clinic	A descriptive cross-sectional study	Patients at ANC	BSE positively associated with attained educational level.
Sama et al, 2017 [55]	Cameroon	Urban	Teacher Training College	Descriptive cross-sectional study	First cycle female undergraduate students	93% recognized the importance of BSE for their health.
Sambo et al, 2013 [50]	Nigeria	Urban	University campus	A cross-sectional descriptive stud	Female students	30.2% mentioned breast lump as a feature of BC, no significant association between knowledge and practice of BSE and level of study.
Sarfo et al, 2013 [58]	Ghana	Urban	University campus	A single case study approach	Female nursing students	The majority had knowledge on BSE, cited BSE as a method of BCS and that BSE was necessary
Segni et al, 2016 [56]	Ethiopia	Urban	University campus	A Cross-sectional study	Female students	44.2% knew BSE is done monthly, 53.8% knew painless nodules as a sign to diagnose BC.

### Study findings

Of the 21 studies, 18 studies reported evidence on knowledge and practice [45–52, 56–65] while 2 studies reported on knowledge only [53, 55], and one study on practice only [54]. Six of the included further reported evidence on the attitude of women towards BSE [49, 52, 56, 58, 60, 63].

### Knowledge of women on BSE

Of the total 20 included studies that presented evidence on women’s knowledge of BSE, eleven reported high (> 70% of study participants) knowledge on BSE [46–48, 50, 58, 59, 61–65], two reported average BSE knowledge (≥ 50% of the study participants) [49, 60], and seven reported low (< 50% study participant) BSE knowledge [45, 51–53, 55–57]. Of the eleven studies that revealed more than 70% of their study participants having knowledge on BSE, four were conducted in Nigeria [46, 50, 62, 63], two each in Ghana [48, 58] and Uganda [47, 59], one each in Cameroon [61], Kenya [64], Sudan [65].

In Cameroon, Nde et al. reported that 73.5% of their study participants had knowledge on BSE of which 37.3% knew BSE is performed monthly, 9% knew how to perform it, and 88.6% knew BSE is important for early detection of BC [61]. In Sudan, Idris et al. study reported 86% BSE knowledge among the participants [65]. Kimani & Muthumbi study in Kenya among female medical students in 2008 reported BSE 94.4% BSE awareness [64].

Fondjo et al. study evaluated the knowledge among senior high school and tertiary students in Ghana and reported 90.9% BSE knowledge among participants, of which 91.6% knew BSE as a tool for early detection of BC, 45.8% knew BSE was done monthly, 21.1% knew BSE is performed after menstruation, and the majority (63%) knew the posture to assume during the performance of BSE [48]. Sarfo et al. also reported 95% BSE knowledge among female nursing students and of which 60% of them knew BSE as a screening method for BC detection [58].

Two studies conducted in Uganda (Obaikol et al. & Godfery et al.) revealed 81.5 and 76.5% BSE knowledge respectively among their participants [47, 59].

In Nigeria, a study reported 98.9% BSE knowledge in Owerri [62]. Casmir et al. reported that the participants who knew the age to begin BSE were 20.5 and 96.7% knew BSE was beneficial [62]. BSE knowledge of 85.1, 82, and 74% was reported by Gwarzo et al.; Faronbi &Abolade; and Sambo et al. among participants in their respective studies [46, 50, 63]. However, Faronbi & Abolade study found that only 22% of the study participants understood the purpose for the performance of BSE, 12% knew BSE is done monthly, and 16% knew the exact age to begin BSE [63]. Nonetheless, two studies involving rural women at a health facility and senior high school students in Nigeria respectively found 52.8 and 56.4% of the participants had access to information on BSE [49, 60]. Isara & Ojedokun, in 2011 reported that 56.4% of the participants had information on BSE, of which, 52.3% knew BSE was a means of screening for BC, 12.5% knew the correct time to perform, and 18.8% knew BSE performance was a monthly required [60].

Of the seven studies that reported evidence of low BSE knowledge among their study participants [45, 51–53, 55–57], four were conducted in Nigeria [51–53, 57], two in Ethiopia [45, 56], and one in Cameroon [55]. Abere et al. assessed the effectiveness of planned teaching program on knowledge and practice of BSE among first-year female midwifery students in Hawassa Health Sciences College [45]. Their study showed that before intervention; 23% had heard of BSE, and 32% knew BSE helps to detect lump early [45]. But after the teaching program, the study found that 100% had heard of BSE, and 96.7% knew BSE facilitates early detection of breast lumps [45]. Makanjuola et al. study also reported that 40% of the study participants had poor knowledge of BSE [52], while 48% of students were reported to have poor knowledge of BSE by Faronbi & Abolade in their study [63]. Similarly, Isara & Ojedokun study aimed at assessing the knowledge of senior high school students on BSE in Nigeria reported that 75.6% of the students had poor knowledge of BSE [60]. Whilst, Segni et al. study among University students found that 91.3% had poor knowledge of BSE [56]. These findings revealed varied knowledge levels on BSE among women in SSA countries, hence, requires further investigations.

### Attitude toward BSE

Six studies out of the 21 included studies reported on the attitude of the participants toward BSE [49, 52, 56, 58, 60, 63]. The study by Isara & Ojedokun in 2011 showed that the majority (82.6%) of senior high school students in Nigeria had a positive attitude toward BSE [60]. Sarfo et al. study also reported that female nursing in Ghana had a positive attitude toward BSE [58]. Whereas in Ethiopia, 59.2% of the study participants were found to have a positive attitude toward BSE [56], and moderate attitude was reported by Nde et al. in Cameroon [61]. Faronbi & Abolade; and Olowokere et al. in their studies in a rural setting in Nigeria reported poor attitude toward BSE [49, 63]. This finding demonstrates limited literature on women’s attitudes towards BSE.

### Practice of BSE

Of the 21 studies, 19 reported evidence on the practice of BSE [45–52, 54, 56–65]. Four studies gave evidence of over 50% of participants who had practiced BSE [46, 50, 54, 57] while 15 studies recorded evidence of low practice [45, 47–49, 51, 52, 56, 58–65]. Agbonifoh, 2016 study among tertiary students in Edo state in Nigeria found out there was a high level of practice of BSE among participants [54]. Similar reports of high (78%) practice of BSE was recorded by Onwere et al. in 2009 among antenatal patients in a teaching hospital in a South Eastern Nigeria [57]. Sambo et al. study in 2013 also reported that 55% of the undergraduate students in Northern Nigeria were practicing BSE [50]. Gwarzo et al. study in 2009 reported that 57% of the participants had ever practiced BSE 32.1% [46].

Of the 15 with a low level of practices, six were conducted in Nigeria [49, 51, 52, 60, 62, 63], two each in Ethiopia [45, 56], Ghana [48, 58], and Uganda [47, 59]; one each was conducted in Cameroon [61], Kenya [64] and Sudan [65]. Gwarzo et al. reported that only 19% of their study participants were currently practicing BSE monthly [46]. Among the rural women in Ala community in Nigeria, 13% of the women practiced BSE as reported by Makanjuola et al. [52]. The study by Casmir et al.in 2015 also reported 32.5% of the participants practiced BSE [62], 31.4% was also reported among senior secondary school students in Abuja Nigeria [60]. Again, in Nigeria, two studies found 12 and 11.7% of the study participants who practiced BSE [49, 63]. The lowest percentage (0.4%) of BSE practice was reported by Obaji et al. in their study in Nigeria involving market women in 2013 [51]. These findings show BSE practice remains a challenge in SSA and further studies are needed to investigate the barriers and facilitators of BSE practice.

## Discussion

We conducted a scoping review to explore evidence on knowledge, attitude, and practice of BSE (KAP) among women in SSA. This study revealed evidence of women’s KAP on BSE in seven SSA countries (Sudan, Nigeria, Ghana, Cameroon, Kenya, Ethiopia, and Uganda). The results generally demonstrate limited published research on knowledge, attitude and practice of BSE among women in SSA. The results also revealed varied knowledge levels on BSE among women in SSA countries. It further suggested that BSE practice remains a challenge in SSA.

We found evidence of women’s KAP on BSE reported in seven SSA countries. This implies that we found no evidence in about 39 countries classified among SSA countries including the following: Angola, Benin, Botswana, Burkina Faso, Burundi, Cape Verde, Central African Republic, Chad, Comoros, Congo, Côte d’Ivoire, the Democratic Republic of the Congo, Equatorial Guinea, Eritrea, Gabon, Gambia, Guinea, Guinea-Bissau, Lesotho, Liberia, Madagascar, Malawi, Mali, Mauritania, Mauritius, Mozambique, Namibia, Niger, Rwanda, Sao Tome and Principe, Senegal, Seychelles, Sierra Leone, South Africa, Swaziland, Togo, United Republic of Tanzania, Zambia, and Zimbabwe although BC may be also be rising in these countries. This finding suggests limited research on knowledge, attitude, and practice of BSE among women in SSA. Similarly, Che Mut et al. systematic review BSE among female students also found limited studies [66]. This is worrying since BSE is a primary screening technique to detect breast abnormalities reporting for CBE or mammography.

We also found knowledge variations on BSE among women in SSA countries. It ranges from a low level to a higher level of knowledge with more of the low level of knowledge been reported. This, therefore, requires an intervention to increase the knowledge level of women in SSA. BSE has been reported as one of the screening methods for early detection of BC [5] therefore, in-depth knowledge, monthly practice and good attitude toward BSE are important for recognition of a normal breast and for detection of any abnormalities which is necessary for the control of morbidity and mortality associated with BC through early diagnosis of BC [16]. It further suggested that BSE practice remains a challenge in SSA. This challenge would require an intensive public campaign to help improve the practice of BSE in SSA.

### Implication for practice

The review study included studies conducted in SSA where most of the clients with BC still present with end-stage of BC [67], thus increasing the morbidity and mortality associated with BC in SSA [5, 68]. BSE is one of the screening methods for early detection of symptoms of BC, even though CBE and mammograms are the most reliable methods. CBE is done at the health facility by trained personnel and mammogram also done at the facility but for clients 40 years and over. This implies that the women will have to go to a facility for any of these screening method and for mammogram at the age of 40 year, for a woman to go to the facility for screening except where mass screening are carried out, she need to have observed an abnormality which will be detected through BSE [69]. Our findings from the studies reviewed showed that some participants were able to detect lumps and other abnormalities in their breast from a regular practice of BSE [46, 47, 61, 65] It implies that planned tutorial on BSE will have a lot of impact especially among the health personnel’s as reported in some of the studies [45, 54, 65] and if the women are taught to practice BSE regularly, knowing what is abnormal in their breast, and any detection will necessitate seeking early medical care since they would have known the consequences of delays in reporting breast abnormalities [51, 58, 60, 65] This study’s findings showed most of the participant indicated that BSE was a form of a screening method for early detection of abnormalities, therefore, an intensive public and institutional education is required on KAP of BSE with the aiming at early detection of abnormalities and subsequently seeking of early medical care, thereby reducing morbidity and mortality associated with BC.

### Implication for research

Our study shows limited published research on BSE in SSA. Most of the studies were conducted among tertiary students in an urban setting indicating a gap in literature among rural women. We hope our study will stimulate research studies on KAP of BSE among rural women in SSA who are more disadvantaged in accessing other CBE and mammography screening methods. We also recommend primary studies to assess KAP in those countries we found no evidence since the prevalence of BC is increasing in SSA. We further recommend primary research to assess the practice of BSE among midwives and nurses who had formal training on BSE during their course of study. We further recommend a systematic review and a meta-analysis to assess the impact of BSE knowledge and practice of BSE in SSA. Knowledge of the factors that influence the practice of BSE may be useful. Moreover, most of the included studies were descriptive cross-sectional surveys. This demonstrates the need for more interventional studies to identify innovative contextualised strategies or approaches to improving the practice of BSE among women in SSA.

### Strengths and limitations

This scoping review probably is the first broad study to map evidence on KAP of BSE among women in SSA countries. The study showed a noteworthy gap in the literature on KAP of BSE among women in SSA countries. This study’s methodology allowed the identification of eligible articles methodically, charting and analysing the outcomes [20]. Nonetheless, this study also has several limitations. This study sought to present recent evidence (within the last 10 year) hence, it included only articles published from 2008 onward. So, it possible some relevant articles published before 2008 were excluded. No quality appraisal was conducted as part of this study, but it is not essential due to the explorative nature of scoping reviews although we planned to assess the methodological quality of the included studies in the published protocol. We realized the number of included studies was few hence, reporting the risk of bias with these few studies may not be useful. Nonetheless, we will endeavour to perform the quality appraisal in the next phase of this study (a follow-up study full systematic review and meta-analysis). We also search for literature in only four databases, but it possible other relevant studies exist in other databases such as Scopus, Web of Science, and EMBASE that were not captured. We recommend future studies to conduct additional searches in those databases that were not captured by this study. It is possible researches on KAP of BSE existed under different terminologies that were not captured in the review. Nevertheless, we included MeSH terms to help address this. Furthermore, a meta-analysis using the quantitative data could generate more information but, this is not essential for scoping review studies. There may be several factors such as religious and cultural beliefs contributing to the KAP of BSE which were not captured by this study. We, therefore, recommend researches on the factors influencing KAP of BSE in SSA.

## Conclusion

This study demonstrated that there is a paucity of published literature on women’s knowledge, practice, and attitude of BSE in SSA. Most of the included studies reported low KAP of BSE. Considering the resource constraints in most health facilities in SSA countries, adequate knowledge and practice, as well as a good attitude towards BSE, is essential. Hence, this study recommends further studies on knowledge, practice, and attitude of BSE, to identify contextual challenges and provide evidence-based solutions to improve women’s knowledge, practice, and attitude of BSE in SSA.

## Supplementary information

**Additional file 1.** Preferred Reporting Items for Systematic reviews and Meta-Analyses extension for Scoping Reviews (PRISMA-ScR) Checklist.

**Additional file 2.** Electronic search results for title screening.

## Data Availability

The data supporting the conclusion of this paper are available through the detailed reference list. No original datasets are present since this is a review of the existing literature.

## Abbreviations

BC: Breast cancer
BSE: Breast self-examination
CBE: Clinical breast-examination
DALYs: Disability-adjusted life years
KAP: Knowledge, attitude, and practice
MMAT: Mixed method quality appraisal tool
LMICs: Low and middle-income countries
PCC: Population, content and context
PRISMA-ScR: Preferred reporting items for systematic reviews and meta-analyses modified for scoping reviews
SDG: Sustainable development goal
SHS: Senior high school
SS: Senior secondary
SSA: Sub-Sahara African
WHO: World health organization
