# Supplementary material for: Women’s knowledge, attitude, and practice of breast self- examination in sub-Saharan Africa: a scoping review
Source: Arch Public Health. 2020 Sep 22;78:84. doi: 10.1186/s13690-020-00452-9 (PMC7507650; doi:10.1186/s13690-020-00452-9)
Supplement: Supplementary file 2 — Additional file 2. Electronic search results for title screening. [file 13690_2020_452_MOESM2_ESM.docx]

**Supplementary file 1:** Electronic search results for title screening

| **Date** | **Databases** | **Keywords** | **Search results** | **Eligible studies** |
| --- | --- | --- | --- | --- |
| 24/05/2019 | PubMed | (((("knowledge"[MeSH Terms] OR "knowledge"[All Fields]) OR ("attitude"[MeSH Terms] OR "attitude"[All Fields])) OR "practice"[All Fields]) AND ("breast self-examination"[MeSH Terms] OR ("breast"[All Fields] AND "self-examination"[All Fields]) OR "breast self-examination"[All Fields] OR ("breast"[All Fields] AND "self"[All Fields] AND "examination"[All Fields]) OR "breast self examination"[All Fields])) OR ("breast self-examination"[MeSH Terms] OR ("breast"[All Fields] AND "self-examination"[All Fields]) OR "breast self-examination"[All Fields] OR ("self"[All Fields] AND "breast"[All Fields] AND "examination"[All Fields]) OR "self breast examination"[All Fields]) AND ("women"[MeSH Terms] OR "women"[All Fields]) OR ("female"[MeSH Terms] OR "female"[All Fields]) AND "Africa"[All Fields] OR "sub sahara africa"[All Fields] OR "south of the sahara"[All Fields] OR "SSA"[All Fields] AND ("2008/01/01"[PubDate] : "2019/05/24"[PubDate]) | 121,885 | 153 |
| 27/05/2019 | Google Scholar | “breast self-examination” OR “self-breast examination” AND “knowledge” OR “attitude” OR “practice” AND “women” OR “female” AND “Africa” OR “sub sahara africa" OR "south of the sahara" OR "SSA" | 2,760 | 99 |
| 28/05/2019 | Science Direct | “breast self-examination” OR “self-breast examination” AND “knowledge” OR “attitude” OR “practice” AND “women” OR “female” AND “Africa” or “sub sahara africa" or "SSA" | 307,789 | 12 |
| 30/05/2019 | CINAHL | SU “knowledge” OR SU “attitudes” OR SU “practice” AND SU “breast self examination” OR SU “self breast exam” AND SU (“women” or “female”) AND SU (“Africa” or “sub sahara africa" or "south of the sahara" or "SSA) | 162,710 | 101 |
| Total |  |  | 595,144 | 365 |
